# Exploring the interplay between food security and antenatal care utilization among pregnant women in Southern Ethiopia: Insights from an institution-based cross-sectional study

**DOI:** 10.1016/j.eurox.2024.100288

**Published:** 2024-02-13

**Authors:** Gemeda Wakgari Kitil, Lema Fikadu Wedajo, Gizu Tola Feyisa, Bekem Dibaba Degefa, Shambel Negese Marami, Agmasie Damtew Walle, Alex Ayenew Chereka, Dagne Deresa Dinagde

**Affiliations:** a Departments of Midwifery, College of Health Sciences, Mattu University, Metu, Ethiopia; b Departments of midwifery, College Medicine and Health Sciences, Wallaga University, Nekemte, Ethiopia; c Departments of Health Informatics, College of Health Sciences, Mattu University, Metu, Ethiopia

**Keywords:** Antenatal care services, Pregnant women, Satisfaction, Ethiopia

## Abstract

**Background:**

Ensuring the satisfaction of pregnant women with antenatal care is crucial for positive pregnancy outcomes and their engagement with emerging technologies and alternative care models. Maintaining high satisfaction during the antenatal period significantly impacts the well-being of both the expectant mother and the unborn child. Despite the recognized importance of antenatal care satisfaction, comprehensive information on satisfaction levels and influencing factors in the specific study area is lacking. Therefore, this study aims to assess antenatal care service satisfaction and associated factors among pregnant women in Arba Minch town, southern Ethiopia.

**Methods:**

We conducted an institution-based cross-sectional study among 418 pregnant women from December 2022 to January 30, 2023, using a systematic sampling method. Data were collected using the Kobo Toolbox and analyzed with SPSS Version 26. The threshold for statistical significance was set at a p-value of less than 0.05.

**Results:**

Out of 418 participants, 54.3% (95% CI=49.4–60.4) expressed satisfaction with antenatal care services. Factors significantly associated with women's satisfaction included: being unable to read and write (AOR=2.37; 95% CI: 1.97–3.80), being aged 25–29 years (AOR=3.20; 95% CI: 1.65–6.22), receiving antenatal care at a hospital (AOR=1.81; 95% CI: 1.05–3.12), having a previous history of antenatal visits (AOR=2.59; 95% CI: 1.26–5.30), a monthly income of 2500–5000 ETB (AOR=1.44; 95% CI: 1.21–3.94), waiting times of less than 30 min (AOR=2.59; 95% CI: 1.52–4.41), maintaining a positive attitude towards antenatal care (AOR=2.50; 95% CI: 1.05–3.65), and having a secure food source (AOR=2.06; 95% CI: 1.13–3.78).

**Conclusion:**

Over 54% of participants were satisfied with antenatal care services. To improve satisfaction levels, recommended strategies include enhancing healthcare infrastructure, establishing maternity waiting areas, reducing waiting times, and expanding services to remote areas.

## Introduction

Preventable maternal diseases and mortality rates remained higher in the period before the establishment of the Sustainable Development Goals. The World Health Organization (WHO) aims to save mothers' lives by ensuring that all pregnant women receive prompt, appropriate, evidence-based, and high-quality prenatal care throughout their pregnancies [1]. To achieve the third Sustainable Development Goal, which is to 'ensure healthy lives and promote well-being for all at all ages,' Sub-Saharan African nations will need to increase the utilization of antenatal care services [2]. While the main objective of a healthcare system is to improve health outcomes, the WHO also emphasizes the importance of goals that enhance the system's responsiveness, such as increasing women's satisfaction with antenatal care services [3].

The World Health Organization (WHO) revised its recommendation in 2016, increasing the minimum number of antenatal care (ANC) visits for pregnant women from four to eight [1]. The level of client satisfaction is determined by the extent to which healthcare providers and services meet the patient's expectations, goals, and preferences [4]. Various models can be employed when implementing, evaluating, and measuring the quality of care. One of the most popular models is Donabedian's logic model, which is divided into structure, process, and outcome components. According to Donabedian, satisfaction refers to how closely a client's experience aligns with their expectations. Client satisfaction is considered one of the crucial metrics for measuring the efficacy of healthcare, indicating the quality of healthcare services [5]. Patient satisfaction serves as an essential measure of accessibility and care quality, both of which reflect the effectiveness of the healthcare system [6]. Patients who receive inadequate medical care are dissatisfied, and dissatisfied patients are less likely to return to the same facility. This has an impact on both the institution's economic health and its organizational performance [7].

The level of satisfaction women experience with ANC (Antenatal Care) services varies according to various research studies. Based on these studies, the satisfaction rates are as follows: approximately 69% in the United States [8], 48% in Myanmar [9], 90% in Kazakhstan [10], 55% in Pakistan [11], 90.0% in Nigeria [12], and 41.1% in Egypt [4].

The utilization of ANC services in many African nations is strongly associated with the satisfaction of the clients. Pregnant women in Ethiopia reported satisfaction levels with ANC services ranging from 21.5% to 83.9%, as shown in several surveys [13], [14]. The satisfaction rates for ANC among pregnant women were as follows: 70.3% in Harari, 68.0% in Arba Minch Zuria, 79.2% in Hawasa, 53.8% in Debre Tabor, 33.0% in Bursa Woreda of Sidama Zone, 21.5% in Denba woreda of Gamo Gofa Zone, 74% in Hossana Town, 60.4% in Jimma, 64.1% in Bedessa town of Wolaita zone, 83.9% in Alganesh Health Center Shire, North West Tigray, and 85.7% in Wolaita zone [13], [14], [15], [16], [17], [18], [19], [20], [21], [22], [23].

Depending on the study setting, women's satisfaction with ANC (Antenatal Care) services is associated with numerous factors. Several variables have been identified, including a woman's previous history of ANC, whether she received ANC services at a hospital, the sex of the service provider, the timing of initiation, the number of ANC visits, a history of abortion, unintended pregnancies, advice on danger signs during pregnancy, respectful maternity care, the family's monthly income, the type of healthcare facility, the type of pregnancy, privacy, cleanliness, respect, distance to the facility, waiting time, counseling during pregnancy, and maternal education [13], [14], [15], [17], [22]. The quality, satisfaction, and utilization of maternal and reproductive healthcare services, particularly ANC, have been significantly impacted by the coronavirus disease (COVID-19). Therefore, it is crucial to regularly monitor and assess the performance of these services [24], [25].

There is a scarcity of information on the satisfaction of pregnant women in the study settings, even though it is crucial for the quality of antenatal care (ANC) to continue improving and for pregnant women to receive high-quality healthcare services. Currently, there are few findings available regarding the levels of satisfaction among pregnant women and the factors associated with it in Ethiopia, particularly in the study area. Most previous research in Ethiopia has focused on maternal satisfaction levels at individual healthcare facilities. Furthermore, other factors, such as food security and pregnant women's attitudes towards ANC satisfaction, have yet to be identified in the specific study area.

Therefore, the objective of this study was to assess antenatal care satisfaction and its associated factors among pregnant women receiving antenatal care in public facilities in Arba Minch town, located in southern Ethiopia, in the year 2023.

## Methods and materials

### Study area and period

The study was conducted in public health facilities in Arba Minch town. The town is located 505 km southwest of Addis Ababa, the capital city of Ethiopia, and 275 km away from Hawassa, the commercial and administrative center of the southern region. According to the 2020 population projection, Arba Minch town has a total population of 108,956, with 55,568 females and 53,388 males. The town has two public hospitals, one private hospital, and two health centers. All of the health facilities are providing perinatal care. There are 15 nurses and 30 midwives providing antenatal care in those health facilities. The public health institutions in the town are expected to serve more than half a million people in the town and nearby districts [26]. Data were collected from December 1, 2022, to January 30, 2023.

### Study population

All mothers who had ANC visits in Arba Minch town public health facilities are available during the data collection period.

### Eligibility criteria

All mothers who had ANC visits in Arba Minch town health facilities and who were willing to give information were included and mothers who were seriously ill and unable to respond were excluded.

### Sample size determination

The sample size was calculated using a single population proportion formula:

$n={\left(\mathrm{za}\right)}^{2}\frac{p(1-p)}{d^{2}}$, considering the following assumptions: prevalence of women’s satisfaction was taken from a study conducted in Debra Tabor town [7] at 53.8%, with a confidence interval of 95% and, a 5% margin of error.

$$n={\left(\mathrm{za}\right)}^{2}\frac{p(1-p)}{d^{2}}$$

$$n={\left(1.96\right)}^{2}\frac{0.538(1-0.538)}{0.05^{2}}=382$$

Finally, the required sample size for this particular study was decided by adding a 10% non-response rate which is 420.

### Sampling technique and procedure

**Fig. 1:**
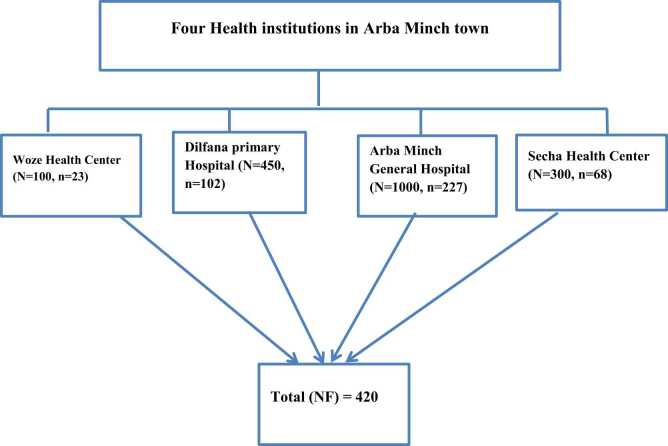
The schematic diagram shows the proportional allocation of a sample size of each health institution in Arba Minch Town 2023.

There are two public hospitals and two public health centers in Arba Minch town that provide curative and preventive services to the local population. The study encompasses all the hospitals and public health centers in the community. The next step involved identifying mothers who regularly visit these health institutions for antenatal care. Using the proportionate probability technique, we calculated the sample size for the two health facilities. The total number of participants was determined based on the antenatal care registration book from the previous year for the same months (i.e., December to January) at all four healthcare facilities. According to the 2021 annual antenatal care report for each health facility, 1000 women from Arba Minch General Hospital, 450 from Dilfana Primary Hospital, 300 women from Secha Health Center, and 100 women with ANC follow-up at Woze Health Center were included as the sampling frame. We then employed systematic random sampling to select the respondents. The first respondent was randomly chosen from the first four women who had received ANC services on the first day of data collection. Subsequently, every fourth woman was selected as a participant, following a systematic random sampling procedure, until we reached the required sample size for the included public health institutions (See Fig. 1).

### Study variables

**Dependent variables:** Women’s satisfaction with ANC services (Satisfied, not satisfied).

### Independent variables

**Socio-demographic factors include** age, educational level, employment status, and occupation, place of residence, husband's education, and family's monthly income.

**Health facility-related factors** encompass insurance status, distance to the health facility, waiting time, counseling on danger signs, and the provision of respectful maternity care.

**Obstetrics-related factors** encompass: type of pregnancy, previous mode of delivery, presence of chronic diseases, type of healthcare institution, knowledge and attitude towards antenatal care (ANC) services, perceived quality of ANC services, food security, parity, partner support, awareness of danger signs, obstetric history, previous ANC visit history, the number of ANC visits, and the number of children**.**

### Operational definitions

#### Women’s satisfaction with ANC services

Women were considered satisfied with antenatal care services if their questionnaire responses on this topic were either higher than or equal to the overall mean satisfaction score. To determine satisfaction, a total score was calculated, and if a woman's score exceeded the mean, she was considered satisfied with the antenatal care she received; otherwise, she was considered unsatisfied [27].

#### Knowledge of ANC

We assessed the overall level of participants' knowledge of prenatal care by assigning a score of 1 for correct responses and 0 for incorrect responses, following the descriptions below. We then calculated a total score and a mean score. A score below the mean indicated poor knowledge, while a score at or above the mean indicated good knowledge [28].

#### Food insecurity

Household food security was assessed using the Household Food Insecurity Access Scale (HFIAS) created by the Food and Nutrition Technical Assistance (FANTA) Programme of the United States Agency for International Development. In this study, a 9-item questionnaire measured household food security, classifying participants as either 'food secure' or 'food insecure' based on their access to sufficient food for an active and healthy lifestyle [9].

**Attitudes toward ANC** were evaluated using the Likert scale, with ratings of 1 for strong agreement, 2 for agreement, 3 for disagreement, and 4 for strong disagreement. Participants received a positive attitude score if their responses to the attitude assessment questions were above the mean and a negative attitude score if their responses were below the mean **[**29].

#### Level of respect and non-abusive care

A woman was classified as having received "disrespected and abused care" if she answered "yes" to all of the questions about her labor. A mother was considered to have a "good level of respect" if she answered "no" to six questions **[**30].

**Types of facilities** include hospitals, health clinics, or health posts that they choose to visit.

#### Waiting time

The waiting time for receiving services is longer if a mother waits for more than 30 min without receiving services in less than 30 min **[**15].

**Partner support** is assessed using an adapted 8-item Spousal Support Scale (SSS), which can be tailored to the local context. The SSS employs reverse scoring, where a score of 5 indicates 'strongly agree,' and 1 indicates 'strongly disagree,' resulting in a range of scores from 8 to 48. A respondent's perception of partner support is considered positive if their score exceeds the mean, indicated by a higher score [31].

## Data processing and analysis

Data collection utilized a structured, pre-tested interviewer-guided questionnaire, which was adapted through a review of relevant literature [15], [16], [17], [20], [22] with subsequent modifications. This questionnaire comprised three sections: socio-demographic factors, obstetrics factors, and health service-related factors, and was designed in both English and Amharic. Participants were selected from women attending ANC services in public health facilities in Arba Minch town using systematic sampling, based on the case flow from the same three months in the previous year as a sampling frame. The data collection was conducted by three midwives with BSc degrees, chosen for their experience and fluency in the local language. Their work was supervised by two midwives holding MSc degrees.

## Data quality management

A data collection method was developed to ensure data quality following an in-depth review of relevant literature and similar studies. After two days of training for data collectors and supervisors, properly designed data collection tools were provided. The questionnaire was pre-tested two months before data collection began among 21 mothers attending their ANC follow-up at Chencha Primary Hospital. All necessary corrections were made based on the pretest results to avoid confusion and ensure better completion of the questions. The maintenance of respondents' privacy and confidentiality, along with effective communication skills gained through training sessions for both data collectors and supervisors, contributed to the study's quality. Each day, all questionnaires were reviewed and checked at the end of the data collection period. Any errors, such as double data entry, missing values, consistency issues, and outliers, were corrected by the supervisor and data collector as necessary.

## Data analysis and entry

The data was coded, collected, cleaned, and entered into the Kobo Toolbox. It was then exported to the Statistical Package for Social Science (SPSS) version 26 for analysis. Inconsistencies and missing values were addressed by conducting various data explorations, including running frequency checks. Descriptive statistics, such as frequency distributions, means, and standard deviations, were computed. Bivariate analysis was primarily conducted to determine which independent variables were associated with the dependent variable. Independent variables with marginal associations (P < 0.25) in the bivariate analysis, those that were biologically plausible, and those that had shown significant associations in previous studies were entered into a multivariate logistic regression analysis to detect their association with antenatal dropout.

Multicollinearity among independent variables was assessed, and the appropriateness of the analysis model was evaluated using the Hosmer-Lemeshow test. Finally, adjusted odds ratios (AOR) with 95% confidence intervals (CIs) were estimated to assess the strength of associations, and statistical significance was declared at a p-value of < 0.05. The results were presented using tables, figures, and text.

### Ethical approval and consent to participate

The study was approved by the Institutional Review Board of Arba Minch University College of Medicine and Health Sciences Ethical Review Board with the ethical approval reference number IRB/1328/2022. An official letter of support was written by the College of Medicine and Health Sciences to the administrators of the Arba Minch town health bureau. Verbal informed consent was obtained from all the participants. To protect confidentiality names and personal identification were not included in the questionnaires. Moreover, the study was conducted based on the declaration of Helsinki.

## Results

### Socio-demographic characteristics of participants

Out of a total of 420 study participants, 418 were interviewed and provided accurate information, resulting in a response rate of 99.5%. Two participants declined to participate in the study as they were in a hurry to leave. Among the participants, the majority, specifically 204 (49%), were housewives, and approximately 163 (39.0%) could read and write. Additionally, 338 (80.8%) of the study participants had a monthly income exceeding five thousand Ethiopian birr (Table 1).

**Table 1:** Socio-demographic characteristics of study participants at Arba Minch town public health facilities Southern Ethiopia, 2023 (n = 418).

**Variables**	**Response**	**Frequency (n)**	**Percent (%)**
Age(in years)	< 20 20-24 25-29 30-34 35 and above	7 91 104 88 128	1.7 21.8 24.8 21.1 30.6
Residence	Rural Urban	125 293	30.0 70.0
Maternal Occupation	Civil servant Farmers Housewife Merchant	82 22 205 109	19.6 5.3 49.0 26.1
Maternal education	Unable to read and write Able to read and write Primary Secondary school & above	100 163 92 63	23.9 39.0 22.0 15.1
Maternal occupation	civil servant Farming housewife Traders	81 23 204 108	19.5 5.5 49.0 26.0
Husband education	Unable to read and write Able to read and write Primary Secondary school & above	88 174 64 92	21.1 41.6 15.3 22.0
Income	< 2500 ETB 2501-5000 ETB > 5000	20 60 338	4.8 14.4 80.8

### Obstetrics related factors

Among the total study participants, the majority (351 or 84%) were multiparous. During the study, 307 (73.4%) of the participants reported that their pregnancies were planned. A significant portion of them, 344 (82.3%), had a history of previous ANC (Antenatal Care) visits. In terms of ANC follow-up, more than half of the women, 59.1% (247), had fewer than the recommended number of ANC visits (<4 visits), while the rest followed the recommended ANC schedule. Approximately thirty-seven (8.9%) had chronic illnesses, such as diabetes mellitus, asthma, and hypertension. Two-hundred forty-one (57.7%) of the study participants exhibited a positive attitude toward their ANC services (Table 2).

**Table 2:** Obstetric-related characteristics of pregnant women attending ANC services at Arba Minch town public health facilities, Southern Ethiopia, 2023 (n = 418).

**Variables**	**Response**	**Frequency (N)**	**Percent (%)**
parity	Primipara Multipara	67 351	16.0 84.0
Types of Pregnancy	Planned Unplanned	307 111	73.4 26.6
mode of the previous delivery	Vaginal Cesarean section	335 83	80.1 19.9
Advice on danger signs of pregnancy	Yes No	370 46	88.9 11.1
History of ANC	Yes No	344 74	82.3 17.7
Number of visits	recommended contact (5–8) less contact (<4)	171 247	40.9 59.1
why less than 8 contact	Discontinue Delay registration 8 visits and above	73 174 171	17.5 41.6 40.9
Knowledge of ANC	Good knowledge Poor knowledge	214 202	51.4 48.6
Women’s Attitude to ANC	Positive Attitude Negative Attitude	241 177	57.7 42.3
Partner support	Positive perception of partner support A negative perception of partner support	234 182	56.3 43.8
Bad obstetric history	Yes No	139 279	33.3 66.7
chronic illness	Yes No	37 381	8.9 91.1
Number of children	2-5 Children > 5 children	393 25	94.0 6.0

### Health services-related factors

Approximately three-fourths (73.5%) of the women attended their ANC services at the hospital. The majority of women (81.4%) traveled for 30–60 min to reach the health facility for their antenatal care services. Nearly 73.4% (307) of the women waited for less than 30 min to receive ANC services. To access medical institutions and receive ANC care, the majority of women (63.9%) who traveled by car or motor vehicle had to pay more than 20 Ethiopian Birr. Only 46 respondents (11.0%) reported that they did not receive advice from medical professionals regarding ANC services during a previous pregnancy. The majority of the women (63.6%) reported they had received non-abusive treatment and a high level of respect. One hundred fifty respondents (36.1%) used horses, mules, or walked to reach the health center (Table 3).

**Table 3:** Facilities related factors on women’s satisfaction among study participants in Arba Minch town public health facilities, south Ethiopia; 2023 (n = 418).

**Variables**	**Response**	**Frequency**	**Percent (%)**
Level of health facility	Hospital Health center	111 308	26.5 73.5
Distance of your home to facilities	≤ 30 min 30-60 min > 1 h	42 340 36	10.0 81.4 8.6
Waiting time	≤ 30 min > 30 min	307 111	73.4 26.6
Cost of transport	≤ 20 ETB > 20 ETB	80 338	19.1 80.9
Maternity care	Respectful maternity care disrespectful maternity care	266 152	63.6 36.4
Mode of transportation	Horse/on foot By car	150 266	36.1 63.9

### Women’s satisfaction with antenatal care services

**Fig. 2:**
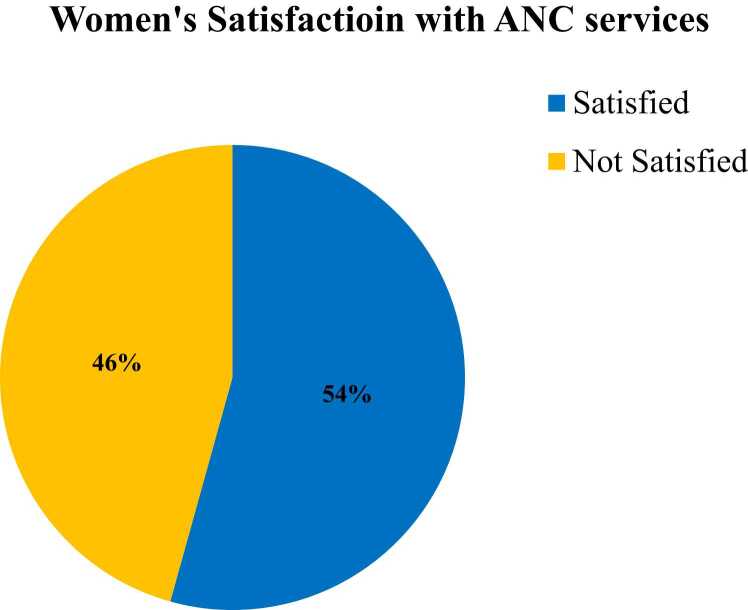
Percentage description of overall women’s satisfaction with the ANC services in Arba town public health facilities, Southern Ethiopia, 2023 (n = 418).

Among the 418 pregnant women participating in the study, 54.3% (95% CI=49.4–60.4) reported satisfaction with the antenatal care services they received (See Fig. 2).

### Factors associated with women’s satisfaction with antenatal care services

The following variables, such as the level of health facility, age, educational level, maternal occupation, family monthly income, distance from health facilities, number of children, planned pregnancy, waiting time to obtain ANC services, history of previous ANC visits, number of ANC visits, chronic illness, attitude towards ANC services, partner support, quality of service, advice on danger signs in pregnancy, knowledge of ANC, respectful maternity care, and food security, were identified as candidate factors for multivariable analysis based on a p-value of ≤ 0.25 in bivariate analysis with a 95% confidence interval.

To determine the variables associated with pregnant women's satisfaction with ANC services, a multivariable logistic regression analysis was conducted. The results indicated that women with an education level unable to read and write had an adjusted odds ratio (AOR) of 2.37 (95% CI: 1.97–3.80) for higher satisfaction. Mothers aged 25–29 years had an AOR of 3.20 (95% CI: 1.65–6.22), and those aged 30–34 years had an AOR of 3.24 (95% CI: 1.62–6.49). Women who received antenatal care at the hospital level had an AOR of 1.81 (95% CI: 1.05–3.12), while those with a history of previous ANC visits had an AOR of 2.59 (95% CI: 1.26–5.30).

Additionally, women with a monthly income of 2500–5000 ETB had an AOR of 1.44 (95% CI: 1.21–2.94), those with waiting times of < 30 min had an AOR of 2.59 (95% CI: 1.52–4.41), and those who received the recommended number of ANC visits (>4 visits) had an AOR of 4.16 (95% CI: 2.44–7.14). Positive attitude towards ANC services was associated with an AOR of 2.50 (95% CI: 1.05–3.65), and women with food security had an AOR of 2.06 (95% CI: 1.13–3.78) for higher satisfaction with ANC services (.

Table 4).

**Table 4:** Multivariable logistic analysis showing factors associated with women’s satisfaction with ANC services in Arba Minch town public health facilities, Southern Ethiopia, 2023(n = 418).

**Variables**	**Response**		**Women’s satisfaction with ANC services**		**COR**	**AOR**
			Satisfied	Not satisfied		
Age category		< 20 20-24 25-29 30-34 35 and above	2 (28.6) 52 (57.1) 51 (49.0) 36 (40.9) 8 (67.2)	5 (71.4) 39 (42.9) 53 (51.0) 52 (59.1) 42 (32.8)	5.11 (.95-27.49) 1.53 (.88-2.68) 2.13 (1.25-3.63) 2.96 (1.69-5.19) 1	2.73 (0.32-22.90) 3.09 (0.43-6.66) 3.20 (1.65-6.22)* 3.24 (1.62-6.49)* 1
Types health facilities		Health center Hospital	41 (36.9) 186 (60.6)	70 (63.1) 121 (39.4)	1 2.6 (1.68-4.11)	1 1.81 (1.05-3.12)
Educational level		Unable to read and write Able to read and write Primary Secondary & above	34 (34.0) 95 (58.3) 52 (56.5) 46 (73.0)	66 (66.0) 68 (41.7) 40 (43.5) 17 (27.0)	5.25(2.6-10.51) 1.94 (1.02-3.66) 2.08 (1.04-4.16 1	2.37 (1.97-3.80)* .964 (.44-2.07) 1.29 (.57-2.94) 1
Family monthly income		< 2500 ETB 2501-5000 ETB > 5000	6 (46.2 26 (44.8 195 (56.2	7 (53.8) 32 (55.2) 152 (43.8)	1.49 (.49-4.55) 1.58 (1.03-2.76) 1	.59 (.15-2.31) 1.44 (1.21-2.94) * 1
History of ANC		Yes No	180(52.3) 47 (63.5)	164 (47.7) 27 (36.5)	1.59 (.94-2.66)	2.59 (1.26-5.30) 1
waiting time		< 30 min > 30 min	185 (60.3) 42 (37.8)	122 (39.7) 69 (62.2)	2.49 (1.59-3.89) 1	2.59 (1.52-4.41)* 1
Number of ANC visits		recommended contact less contact	136 (79.5) 91 (36.8)	35 (20.5) 156 (63.2)	6.67 (4.24-10.4) 1	4.16 (2.44-7.14) 1
Attitude towards ANC		Positive Attitude Negative Attitude	155 (64.3) 72 (40.7)	86 (35.7) 105 (59.3)	2.63 (1.80-4.00) 1	2.50 (1.05-3.65) 1
Food security		food secure food insecure	85 (77.3) 142 (46.1)	25 (22.7) 166 (53.9)	3.97 (2.41-6.55) 1	2.06 (1.13-3.78) 1
**ANC**= antenatal care						

## Discussion

The purpose of the study was to identify modifiable factors associated with pregnant women's satisfaction with antenatal care (ANC) services at public health facilities in Arba Minch town, southern Ethiopia. The overall finding of this study indicated that 54.3% of women (95% CI=49.4–60.4) were satisfied with the ANC services. This finding is consistent with studies conducted in Malaysia (56.7%), Oman (59%), Jimma Town (60.4%), Bahir Dar (52.3%), and Debre Tabor (53.8%) [16], [20], [27], [32], [33]. ‬‬‬‬‬‬‬‬‬‬‬‬‬‬‬‬‬‬‬‬‬‬‬‬‬‬‬‬‬‬‬‬‬‬‬‬‬‬.

However, this study's result is lower than the satisfaction rates found in Thailand (71.8%), Tigray (83.9%), Wolaita zone (82.9%), Hawassa City (79.2%), Hosanna town (74.0%), Arba Minch Zuria (68.0%), west Guji zone (66.0%), and Bedessa woreda of Wolaita zone (64.1%) [13], [15], [17], [18], [22], [34], [35], [36]. On the other hand, it is higher than the satisfaction rates reported in West Shewa (46.7%), Gojam (30.3%), and Demba Gofa woreda (21.5%) [21], [37], [38]. The variation in satisfaction levels can be attributed to the subjective nature of the topic. While most literature measures satisfaction with a basic yes/no response, accurate assessment requires standardized scales and tools. Additionally, differences in study duration may be linked to changing patient expectations due to technological advancements and shifts in attitudes and lifestyles.

Pregnant women who were unable to read and write were twice as likely to be satisfied compared to those with a secondary school education or higher. This finding is consistent with various studies conducted in Ethiopia, including the Harari region, Hossana town, Jimma town, Bursa district in Sidama, and Malaysia [13], [14], [16], [32]. However, this result contrasts with studies from Nigeria and Nepal [12], [39]. This difference may be attributed to women with higher levels of education expecting higher-quality care, as they are more informed about the services and have higher expectations.

Similarly, pregnant women aged 24–29 and 30–34 were three times more likely to express satisfaction with ANC services compared to those aged 35 or older, respectively. This finding aligns with previous studies conducted in Hossana town and Debre Tabor [17], [20].

Pregnant women who received antenatal care at hospitals were twice as likely to be satisfied with their services as compared to those who attended health centers. This difference may be attributed to the greater resources available in hospitals, including improved infrastructure, more personnel, advanced medical equipment, and supplies.

In this study, pregnant women with family incomes between 2501 and 5000 ETB per month showed a significantly stronger association with satisfaction regarding antenatal care services than pregnant women with family incomes greater than 5000 ETB per month. This finding aligns with previous research conducted in Malaysia [32] and Ethiopia [18], [40] which indicated that lower-income groups reported higher levels of satisfaction or a positive correlation between higher satisfaction and minimal out-of-pocket expenses for ANC services. One possible explanation is related to financial expectations: Lower-income pregnant women may have more modest financial expectations and, consequently, be more content with the antenatal care services available within their income range. They might have lower expectations and be less critical of the care they receive, as long as they perceive the services as valuable and appropriate for their budget. On the other hand, pregnant women with higher wealth could have elevated expectations for the quality of care they anticipate. They may be more inclined to express dissatisfaction if the services do not meet their expectations.

Pregnant women who had attended two or more previous antenatal care (ANC) visits were twice as likely to be satisfied with ANC services compared to those women who had no previous ANC visits or only one previous visit. This finding aligns with previous studies conducted in Ethiopia and Nigeria [12], [18]. This might be attributed to the increased awareness and knowledge of pregnant women resulting from frequent ANC visits. The repetition of ANC visits not only fulfills the client's needs but also enhances the effectiveness of meeting those needs.

In comparison to pregnant women with fewer than or equal to four ANC visits, those with five or more visits were four times more likely to be satisfied with the services they received. Studies carried out in the Demba Gofa Woreda and Harari regions showed similar findings [13], [36]. The strong association observed may be due to frequent client visits, which help healthcare providers better understand the significance of ANC, as well as the increasing demands of clients and their overall satisfaction. A satisfied woman is also more likely to adhere to antenatal care recommendations.

Pregnant women who received ANC services within 30 min were 2.6 times more likely to be satisfied than those who waited for 30 min or longer. This study aligns with previous research conducted in the Harari region, Hawassa town, and Jimma [13], [15], [41]. It is possible that pregnant women who live near the medical center are more satisfied because they have convenient access to the facility.

Similarly, pregnant women with positive attitudes toward their ANC services were twice as likely to be satisfied with the antenatal care they received compared to their counterparts. This finding contrasts with the results of a South African study, which reported that most pregnant women were unaware of the benefits and held negative attitudes toward the quality of maternal health provided during ANC services [42]. This study identifies a previously unexplored factor influencing women's satisfaction with antenatal care services.

Furthermore, pregnant women from families with secure access to food had a twofold higher likelihood of being satisfied compared to those from families without such security. This finding has not been reported in previous studies. The dissatisfaction of food-insecure women with antenatal care services may be attributed to their focus on coping with food shortages rather than seeking medical care. Due to food shortages, they may have skipped meals or reduced meal portions, leaving them too weak to travel on foot to a healthcare facility for antenatal care [43], [44].

### Strengths and Limitations of the Study

By safeguarding the privacy of the respondents, we minimized the potential for bias. This study primarily focuses on women's satisfaction with antenatal care services, which is generally enhanced by a better understanding of pregnancy danger signs, the practice of respectful maternity care, planned pregnancies, and regular ANC visits.

However, a limitation of this study is that it relied solely on self-reports from pregnant women; no direct observations were conducted during their care, potentially leading to underreporting or overreporting. Furthermore, the absence of qualitative research to support the findings in this cross-sectional study is another notable limitation.

## Conclusion and recommendations

In this study, the satisfaction of pregnant women with antenatal care services was rated as moderate, with approximately half expressing contentment. Various factors significantly contributed to women's satisfaction. Notably, factors positively associated with satisfaction included: being unable to read and write, age between 25–29 years, receiving antenatal care at a hospital, having a history of previous antenatal visits, having low monthly income, shorter waiting times (less than 30 min), maintaining a positive attitude towards antenatal care, and having a secure food source.

We recommend that stakeholders and healthcare facilities pay appropriate attention to enhancing timely response, focus on improving the infrastructure of healthcare institutions, provide maternity waiting areas, reduce prolonged waiting times for antenatal care, and expand hospital services to remote areas. Additionally, to encourage pregnant women to complete the antenatal care follow-up service, greater efforts are needed to scale up the provision of client-focused antenatal care services. Furthermore, to address other hidden factors that impact client satisfaction in various ways, we recommend conducting an in-depth and well-designed study that employs both quantitative and qualitative approaches.

## Author contributions

Gemeda Wakgari, Gizu Tola, and Dagne Deresa made substantial contributions to the conception and design, study selection, data curation, analysis, interpretation, funding acquisition, investigation, and methodology. They were also involved in the original draft preparation, provided resources, software, supervision, and validation, and contributed to the visualization and review of the study. Agmasie Damtew, Bekam Dibaba, Lema Fikadu, Shambel Negese, and Alex Ayenew wrote the final manuscript draft. All authors significantly contributed to the study's conception and conceptualization. The final work was reviewed, edited, and approved by all authors.

## Funding

No funding was received for this study.

## CRediT authorship contribution statement

**Wedajo Lema Fikadu:** Writing – review & editing, Visualization, Validation, Supervision, Data curation. **Feyisa Gizu Tola:** Writing – review & editing, Visualization, Supervision, Investigation, Formal analysis, Data curation. **Degefa Bekem Dibaba:** Visualization, Validation, Supervision, Conceptualization. **Marami Shambel Negese:** Writing – review & editing, Validation, Methodology, Investigation, Conceptualization. **Kitil Gemeda Wakgari:** Writing – review & editing, Writing – original draft, Visualization, Validation, Supervision, Software, Resources, Project administration, Methodology, Investigation, Funding acquisition, Formal analysis, Data curation, Conceptualization. **Walle Agmasie Damtew:** Visualization, Supervision, Resources, Data curation, Conceptualization. **Chereka Alex Ayenew:** Writing – review & editing, Supervision, Methodology, Data curation, Conceptualization. **Dinagde Dagne Deresa:** Visualization, Resources, Methodology, Formal analysis, Data curation.

## Declaration of Competing Interest

I, Gemeda Wakgari, declare that I have no conflicts of interest regarding the submission of the manuscript titled "*Exploring the Interplay between Food Security and Antenatal Care Utilization among Pregnant Women: Insights from an Institution-Based Cross-Sectional Study in Southern Ethiopia*" to the European Journal of Obstetrics & Gynecology and Reproductive Biology: X.

This research was conducted with the highest standards of integrity and objectivity, and no external funding or competing interests have influenced the design, execution, or reporting of the study. I confirm that I, along with my co-authors, have full control over the data presented in this manuscript and that the findings and conclusions are based on a rigorous and unbiased analysis.

Furthermore, I disclose that there are no financial associations, direct or indirect, or any other situations that might raise questions of bias in the work submitted. I affirm that all sources of financial support for this research have been disclosed in the manuscript.

In the event that any potential conflict of interest arises during the review process, I commit to promptly disclosing such information to the editorial board of the European Journal of Obstetrics & Gynecology and Reproductive Biology: X.

I understand that accurate and transparent reporting of conflicts of interest is essential to uphold the integrity and credibility of scientific research. This declaration is made in the interest of full transparency and adherence to the ethical standards of the journal.

## References

[1] World Health Organization (2016).

[2] Darmoul D., Baricault L., Sapin C., Chantret I., Trugnan G., Rousset M. (1991). World Health Organization. Health in 2015: from MDGs, millennium development goals to SDGs, sustainable development goals. Experientia.

[3] WHO W. Monitoring the Building Blocks of Health Systems: a Handbook of Indicators and. 2010;1–92.

[4] Isma N.I.A.A., Essa R.M. (2017). Pregnant Women’s Satisfaction with the Quality of Antenatal Care At Maternal and Child Health Centers in El-Beheira Governorate. IOSR J Nurs Heal Sci.

[5] Davenport R.J., Dennis M.S. (1996). Assessing the quality of care. Bmj.

[6] Stepurko T., Pavlova M., Groot W. (2016). Overall satisfaction of health care users with the quality of and access to health care services: A cross-sectional study in six Central and Eastern European countries. BMC Health Serv Res [Internet].

[7] Pohwah K., Pui-Mun L., Dhanjoo G. (2006). Impact of deficient healthcare service quality. TQM Mag.

[8] Flacking R., Dykes F. (2017). Perceptions and experiences of using a nipple shield among parents and staff – an ethnographic study in neonatal units. BMC Pregnancy Childbirth [Internet].

[9] Coates J., Swindale A., Bilinsky P. Household Food Insecurity Access Scale (HFIAS) for measurement of food access: indicator guide: version 3. Washington, DC Food Nutr Tech …. 2007;(August):Version 3.

[10] Dauletyarova M.A., Semenova Y.M., Kaylubaeva G. (2018;). Are Kazakhstani Women Satisfied with Antenatal Care. Implement WHO Tool Assess Qual Antena Serv.

[11] Razzaque M.A., Hussain S., Haroon R., Wahid A., Razzaque A. Level of maternal satisfaction with antenatal care and its associated factors in. 2022;47(3).

[12] Onyeajam D.J., Xirasagar S., Khan M.M., Hardin J.W., Odutolu O. Antenatal care satisfaction in a developing country: a cross-sectional study from Nigeria. 2018;1–9. 10.1186/s12889-018-5285-0 PMC5859482 29554885

[13] Simon Birhanu, Melake Demena, Yohannes Baye, Assefa Desalew B.D., GE, Egata G. (2020). Pregnant women ’ s satisfaction with antenatal care services and its associated factors at public health facilities in the Harari region, Eastern Ethiopia. SAGE Open Med.

[14] Dasa T.T. (2017). Maternal antenatal care service satisfaction and factors associated with rural health centers, Bursa District, Sidama Zone, Southern Ethiopia: a cross-sectional study. Journal of Women’s Health Care. J Women’s Health Care.

[15] Lire T., Megerssa B., Asefa Y., Hirigo A.T. Antenatal care service satisfaction and its associated factors among pregnant women in public health centres in Hawassa city, Southern Ethiopia. 2021;

[16] Chemir F., Alemseged F., Workneh D. Satisfaction with focused antenatal care service and associated factors among pregnant women attending focused antenatal care at health centers in Jimma town, Jimma zone, South West Ethiopia; a facility based cross-sectional study triangulated with qua. 2014;1–8. 10.1186/1756-0500-7-164 PMC3994781 24646407

[17] Kebede D.B., Belachew Y.B., Selbana D.W. Maternal Satisfaction with Antenatal Care and Associated Factors among Pregnant Women in Hossana Town. 2020;2020. 10.1155/2020/2156347 PMC7407034 32775404

[18] Lakew S., Ankala A., Jemal F. (2018). Determinants of client satisfaction to skilled antenatal care services at Southwest of Ethiopia: a cross-sectional facility based survey.

[19] Yoseph M., Abebe S.M., Mekonnen F.A., Sisay M. Institutional delivery services utilization and its determinant factors among women who gave birth in the past 24 months in Southwest Ethiopia. 2020;9:1–10. 10.1186/s12913-020-05121-9 PMC7106731 32228558

[20] Ayalew M.M., Nebeb G.T., Bizuneh M.M., Dagne A.H. Women ’ s Satisfaction and Its Associated Factors with Antenatal Care Services at Public Health Facilities: A Cross-Sectional Study. 2021;279–286. 10.2147/IJWH.S293725 PMC7935336 33688266

[21] Mekonnen N., Berheto T.M., Ololo S., Tafese F. Quality of Antenatal Care Services in Demba Gofa Woreda, Gamo Gofa Zone, iMedPub Journals Quality of Antenatal Care Services in Demba Gofa Woreda, Gamo Gofa Zone, Rural Ethiopia. 2017;(December).

[22] Gelaw K.A., Gebeyehu N.A. Maternal satisfaction and associated factors among pregnant women attended at antenatal care service in Bedessa Health Center, Wolaita zone, Ethiopia, 2018. Science Research. 2020;8(2):39–44. 2020;8(2):39–44.

[23] Yohannes B., Tarekegn M., Paulos W. (2013). Mothers’ utilization of antenatal care and their satisfaction with delivery services in selected public health facilities of Wolaita Zone, Southern Ethiopia. Int J Sci Technol Res.

[24] Tadesse E. Antenatal Care Service Utilization of Pregnant Women Attending Antenatal Care in Public Hospitals During the COVID-19 Pandemic Period. 2020;1181–1188. 10.2147/IJWH.S287534 PMC7737544 33335430

[25] Banke-Thomas A., Semaan A., Amongin D., Babah O., Dioubate N., Kikula A., Nakubulwa S., Ogein O., Adroma M., Adiga W.A.D.A. (2022). A mixed-methods study of maternal health care utilisation in six referral hospitals in four sub-Saharan African countries before and during the COVID-19 pandemic. BMJ Glob Health.

[26] Alemu S.S., Ketema T.G., Tessema K.F., Feyisa J.W., Yimer A.A., Kebede B.N. (2022). Preference of homebirth and associated factors among pregnant women in Arba Minch health and demographic surveillance site, Southern Ethiopia. PLoS One [Internet].

[27] Ejigu T., Woldie M., Kifle Y. (2013). Quality of antenatal care services at public health facilities of Bahir-Dar special zone, Northwest Ethiopia. BMC Health Serv Res.

[28] Afaya A., Azongo T.B., Dzomeku V.M., Afaya R.A., Salia S.M., Adatara P. (2020). Women’s knowledge and its associated factors regarding optimum utilisation of antenatal care in rural Ghana: a crosssectional study. PLoS One [Internet].

[29] Girmaye E., Mamo K., Ejara B., Wondimu F., Mossisa M. (2021). Assessment of knowledge, attitude, and practice of skilled assistance seeking maternal healthcare services and associated factors among women in West Shoa Zone, Oromia Region, Ethiopia. Nurs Res Pract.

[30] Worku D., Teshome D., Tiruneh C., Teshome A., Berihun G., Berhanu L. (2021). Antenatal care dropout and associated factors among mothers delivering in public health facilities of Dire Dawa Town, Eastern Ethiopia. BMC Pregnancy Childbirth.

[31] Scale C.G., Guidubaldi J., Cleminshaw H.K. (1989). Development and validation of the. the second handbook on parent education: contemporary. Perspectives.

[32] Pitaloka D.S., Rizal A.M. Patient’s satisfaction in antenatal clinic hospital Universiti Kebangsaan Malaysia. Jurnal Kesihatan Masyarakat (Malaysia). 2006;12(1):1–0. J Community Health.

[33] Ghobashi M., Khandekar R. Satisfaction among Expectant Mothers with Antenatal Care Services in the Musandam Region of Oman‬. 2008;8(3):325–332. PMC3074854 21748079

[34] Prescott L.M. (2003). Highlights of the 43rd interscience conference on antimicrobial agents and chemotherapy (ICAAC). P T.

[35] Fseha B. (2019). Assessment of mothers level of satisfaction with antenatal care services provided at Alganesh Health Center Shire, North West Tigray, Ethiopia. Biomed J Sci Tech Res.

[36] Selgado M.B., Dukele Y.H., Amamo D.D. (2019). Determinants of focused antenatal care service satisfaction in public health facilities in Ethiopia 2018: a mixed study design. J Public Health Epidemiol.

[37] Bekele G.G., Seifu B., Roga E.Y. (2023). Determinants of maternal satisfaction with focused antenatal care services rendered at public health facilities in the West Shewa Zone, Central Ethiopia: a multicentre cross-sectional study. Front Glob Women’s Heal.

[38] Emiru A.A., Alene G.D., Debelew G.T. (2020). Women’s satisfaction with the quality of antenatal care services rendered at public health facilities in Northwest Ethiopia: The application of partial proportional odds model. BMJ Open.

[39] Joshi C., Torvaldsen S., Hodgson R., Hayen A. (2014). Factors associated with the use and quality of antenatal care in Nepal: a population-based study using the demographic and health survey data. BMC Pregnancy Childbirth.

[40] Hailu G.A., Weret Z.S., Adasho Z.A., Eshete B.M. (2022). Quality of antenatal care and associated factors in public health centers in Addis Ababa, Ethiopia, a cross-sectional study. PLoS One [Internet].

[41] Abate T.M., Salgedo W.B., Bayou N.B. (2015). Evaluation of the quality of antenatal care (ANC) service at higher 2 health center in Jimma, South West Ethiopia. OALib.

[42] Drigo L., Luvhengo M., Lebese R.T., Makhado L. (2020). Attitudes of pregnant women towards antenatal care services provided in primary health care facilities of mbombela municipality, Mpumalanga Province, South Africa. Abstract.

[43] District G., Kotiso G.B., Abame D.E., Belachew T., Tamrat M., Ermias D., et al. Disparities in antenatal care service utilization among food secure and food insecure women in Gombora. 2020;37(377):1–11. 10.11604/pamj.2020.37.377.19862 PMC7992396 33796190

[44] Zeleke E.A. Food Insecurity Associated with Attendance to Antenatal Care Among Pregnant Women: Findings from a Community-Based Cross-Sectional Study in Southern Ethiopia. 2020;1415–1426. 10.2147/JMDH.S275601 PMC7646405 33173303

